# Properties of Markov Chain Monte Carlo Performance across Many Empirical Alignments

**DOI:** 10.1093/molbev/msaa295

**Published:** 2020-11-13

**Authors:** Sean M Harrington, Van Wishingrad, Robert C Thomson

**Affiliations:** School of Life Sciences, University of Hawai‘i, Honolulu, HI

**Keywords:** mixing, I + Γ, I + G, parallel tempering, MCMC, MC3, substitution model averaging, MrBayes

## Abstract

Nearly all current Bayesian phylogenetic applications rely on Markov chain Monte Carlo (MCMC) methods to approximate the posterior distribution for trees and other parameters of the model. These approximations are only reliable if Markov chains adequately converge and sample from the joint posterior distribution. Although several studies of phylogenetic MCMC convergence exist, these have focused on simulated data sets or select empirical examples. Therefore, much that is considered common knowledge about MCMC in empirical systems derives from a relatively small family of analyses under ideal conditions. To address this, we present an overview of commonly applied phylogenetic MCMC diagnostics and an assessment of patterns of these diagnostics across more than 18,000 empirical analyses. Many analyses appeared to perform well and failures in convergence were most likely to be detected using the average standard deviation of split frequencies, a diagnostic that compares topologies among independent chains. Different diagnostics yielded different information about failed convergence, demonstrating that multiple diagnostics must be employed to reliably detect problems. The number of taxa and average branch lengths in analyses have clear impacts on MCMC performance, with more taxa and shorter branches leading to more difficult convergence. We show that the usage of models that include both Γ-distributed among-site rate variation and a proportion of invariable sites is not broadly problematic for MCMC convergence but is also unnecessary. Changes to heating and the usage of model-averaged substitution models can both offer improved convergence in some cases, but neither are a panacea.

## Introduction 

Bayesian methods have become extremely popular in phylogenetic inference. This is driven by their ability to efficiently explore complex model spaces, to naturally account for uncertainty, and to allow for rigorous and coherent model selection using marginal likelihoods (Rannala and Yang 1996; Yang and Rannala 1997; Huelsenbeck et al. 2001). The posterior distribution is typically the primary object of interest in Bayesian phylogenetic analyses. This cannot be calculated analytically due to the intractability of computing the marginal likelihood that makes up the denominator of Bayes’ theorem. Therefore, most methods approximate the posterior distribution numerically using Markov chain Monte Carlo, or MCMC (Metropolis et al. 1953; Hastings 1970; Rannala and Yang 1996; Yang and Rannala 1997). A well-behaved Markov chain will sample values for parameters of the model in proportion to their posterior probabilities. Adequately sampling from the posterior distribution requires that a chain has successfully moved from its starting parameter values, which are typically chosen randomly and have relatively low likelihoods as a result, to regions of parameter space with relatively higher likelihoods. This initial movement from starting values to the chain’s stationary distribution constitutes the burn-in phase of MCMC. Second, the chain must mix efficiently and/or run long enough for it to draw an adequate number of samples to closely approximate the posterior. Markov chains of finite length are generally not guaranteed to meet these criteria and so are not generally guaranteed to provide an accurate approximation of the posterior. Because the true posterior distribution is typically unknown, users of MCMC-based methods are therefore faced with the interesting (or maddening, depending on your perspective and particular relationship with MCMC) problem of deciding if chains have performed well enough to produce reliable estimates.

Heuristic diagnostics have been developed to help deal with the problem of identifying whether chains have performed well. Each of these diagnostics seeks to summarize the behavior of one or more Markov chains and provide a statistic that can be used to determine if the chains in question have failed to converge. Just as failure to reject a null hypothesis using a frequentist statistical test does not imply that the null hypothesis is an accurate representation of reality, failure to identify a lack of convergence does not indicate that any particular MCMC analysis has adequately converged. Passing these diagnostic tests increases our confidence that chains are well behaved, but as is so often the case in life, there are no guarantees. Methods for assessing MCMC performance can be broadly grouped into two categories: 1) those that focus on parameter distributions within a single chain, which we refer to as single-chain diagnostics, and 2) those that evaluate congruence among multiple chains, which we refer to as multichain diagnostics. Single-chain diagnostics seek to determine that a chain is sampling stably from a distribution without high levels of autocorrelation, trends, or large, infrequent jumps. This stable sampling is often referred to as stationarity and reflects chains that are mixing well and exploring parameter space efficiently. Multichain diagnostics seek to determine that independent Markov chains starting from different initial values have reached stationarity in the same region of parameter space. This provides evidence that the chains are adequately sampling from the stationary distribution rather than stochastically becoming trapped in local optima, although we reiterate that no diagnostic or combination of diagnostics can guarantee convergence (Huelsenbeck et al. 2002).

In phylogenetics, effective sample size (ESS; Ripley 1987; Neal 1993; Kass et al. 1998) is probably the single most commonly used diagnostic due to its inclusion in the popular Tracer program (Rambaut et al. 2018) associated with the BEAST software (Drummond and Rambaut 2007; Bouckaert et al. 2014). ESS is a single-chain diagnostic that measures the number of independent samples that would produce an equivalent estimate for a parameter distribution as the autocorrelated samples from an MCMC run (Drummond et al. 2006). As MCMC samples are autocorrelated to some degree, the ESS for a parameter will typically be less than the full number of MCMC samples and can be much less if a chain is not mixing well. The typical goal is to achieve a large enough number of independent samples per generation, that is, a high ESS, so that the posterior distribution can be accurately approximated using a relatively short Markov chain. Phylogeneticists often consider ESS to be high enough if they are above an arbitrary cutoff of 200 (Drummond et al. 2006). ESS is typically calculated for continuously valued parameters but has also been adapted for tree topology by using tree distances in calculations (Lanfear et al. 2016). Additional single-chain diagnostics exist for MCMC applications more broadly, but several of these have not or have only rarely been applied to phylogenetic applications of MCMC (reviewed by Brooks and Roberts [1998]).

Common multichain diagnostics applied to phylogenetic MCMC include the potential scale reduction factor (PSRF), sometimes referred to as the Gelman and Rubin statistic or R̂ (Gelman and Rubin 1992), and average standard deviation of split frequencies (ASDSF; Lakner et al. 2008), both of which are reported by default in the popular program MrBayes (Huelsenbeck and Ronquist 2001; Ronquist et al. 2012). The PSRF seeks to assess if continuous parameters from multiple chains have reached the same distribution by comparing the ratio of variance among chains to variance within chains. This ratio decreases toward 1.0 as variance among chains approaches the variance within chains. A high PSRF considerably above 1.0 (variance among chains exceeds the variance within chains) indicates that the chains are sampling from different areas of parameter space and have not adequately converged. The ASDSF is specifically designed to determine if tree topologies are consistent among independent chains. ASDSF is calculated by enumerating all bipartitions in topologies sampled by multiple chains, calculating the frequency at which each split appears within each chain, taking the standard deviation of this frequency across chains, then averaging these standard deviation values (usually focusing only on splits that occur at some frequency above a threshold, typically 0.1). When splits defining the same sets of clades are identified among multiple runs at similar frequencies, ASDSF approaches 0 and is commonly considered to be low enough when it has fallen below 0.01 (Lakner et al. 2008). The “Are We There Yet?” web interface, more recently adapted into the R package “R We There Yet?” (RWTY), introduced a straightforward way to additionally calculate the correlation of split frequencies among chains, another way to examine topological convergence for multiple chains (Nylander et al. 2008; Warren et al. 2017). Like single-chain diagnostics, multichain diagnostics are not guaranteed to detect convergence problems.

The importance of convergence is widely recognized among phylogeneticists, and most users of Bayesian phylogenetic programs have probably struggled to get an analysis to perform adequately. Several aspects of phylogenetic inference can make this a challenging problem. Some parameters in phylogenetic models are inherently correlated with each other (e.g., branch lengths, base frequencies, and substitution rates), parameters may follow complex, multimodal statistical distributions, and the tree topology itself, often the aspect of the model that we are most interested in, is less a parameter than a part of the model’s structure, but one that proposals act on through the MCMC procedure. The tree topology is also a discrete and unordered component of the model that is not easily summarized by means and standard deviations in the way that continuous parameters are. Therefore, standard expectations derived from simpler statistical scenarios of how MCMC diagnostics will perform are not necessarily applicable to phylogenetic MCMC.

Most of our knowledge about how well phylogenetic MCMC analyses converge has been derived from a set of studies focusing on simulated data or a small set of frequently used empirical data sets, and often focus primarily on a single aspect of MCMC performance, such as topology proposals and the exploration of tree space (e.g., Höhna et al. 2008; Lakner et al. 2008; Ronquist and Deans 2010; Höhna and Drummond 2012; Whidden and Matsen 2015; Brown and Thomson 2018). These studies provide useful snapshots of how MCMC performs, including the expectation that convergence will be harder to achieve for larger data sets that explore more complex tree landscapes (e.g., Lakner et al. 2008; Whidden and Matsen 2015; Zhang et al. 2020). Additionally, several authors have discussed the potential for parameters to interact and negatively affect inference or MCMC performance, particularly with regard to models that combine related parameters such as a proportion of invariable sites with Γ-distributed among-site rate variation (often referred to as I + Γ models) (Sullivan et al. 1999; Mayrose et al. 2005; Yang 2006). Parameter interactions such as this have been variously viewed as potentially pathological, merely unnecessary, or of little concern, but have not yet been systematically studied on a large scale.

Although previous studies have provided valuable information about phylogenetic MCMC performance, the field still lacks a broad survey of how well our most commonly used MCMC-based analyses perform for empirical data. This gap leaves researchers to rely on the intuition that they have gained through their own analyses (which may amount to only several dozen across the course of a career) and discussions with others, which means that many seemingly fundamental questions are, so far, unanswered: How frequent are convergence and mixing problems for empirical data sets? Do particular aspects of an alignment predict that MCMC performance will be a challenge? Should we be worried about I + Γ?

We took steps toward answering several such questions by compiling a data set of MCMC output from >18,000 empirical phylogenetic analyses derived from a mixture of earlier studies and new analyses carried out here. In order to match the most common practices in phylogenetics, we employed commonly used analysis settings (e.g., two to four independent runs each with four Metropolis-coupled chains) for widely used Bayesian phylogenetic inference software (MrBayes), and assessed convergence using the most commonly employed diagnostics and thresholds. We used this data set to identify broad trends in phylogenetic MCMC performance. This included characterizing the performance of MCMC across the analyses according to various convergence diagnostics, examining relationships among different diagnostics and their relationships to data set properties, investigating the impact of correlations among parameters, and attempting to improve the performance of poorly performing analyses. We reiterate that all convergence diagnostics are heuristics that can highlight convergence issues but cannot guarantee that they are absent. This is the case for all phylogenetic MCMC where the true posterior is unknown, and so the field must rely on convergence diagnostics to provide useful information if we are to consider any analyses reliable. Broadly, our results highlight several best practices: Researchers should use all available convergence diagnostics, and specifically a combination of single and multichain diagnostics that focus on both topology and non-topology parameters. We also find that poor MCMC performance usually needs to be remedied on a case by case basis by determining the particular cause of the problem. We make several recommendations for increasing the reliability of analyses and provide a large data set that will be useful for future work exploring additional aspects of MCMC performance.

## Results and Discussion

### General MCMC Performance Properties

We began by categorizing analyses based on whether they pass or fail performance thresholds of commonly used diagnostics. Considering each diagnostic independently (i.e., rather than whether analyses pass multiple diagnostics simultaneously), we found that 99% of analyses achieved ESS ≥ 200 for all non-topology parameters, >99% of analyses had correlations among split frequencies across chains ≥0.9, 98% had PSRFs < 1.02, and 97% achieved approximate topological ESS ≥ 200 (table 1). By contrast, only 37% of chains achieved average standard deviations of split frequencies of <0.01. Given the prevalence of ESS in determining whether a single chain has run long enough, we focused considerably on the properties of ESS across parameters and chains. Most parameters reached an ESS above 200 by 5 or 6 million generations, and all parameters show a similar distribution of when this threshold is reached (supplementary fig. S1, Supplementary Material online).

**Table 1.** Proportion of Chains Passing Common Thresholds for Convergence Diagnostics.

ESS > 200	ASDSF < 0.01	Split Freq. Correlation > 0.9	PSRF < 1.02	Topological Approx. ESS > 200
0.988	0.375	0.997	0.980	0.973

**Fig. 1.**
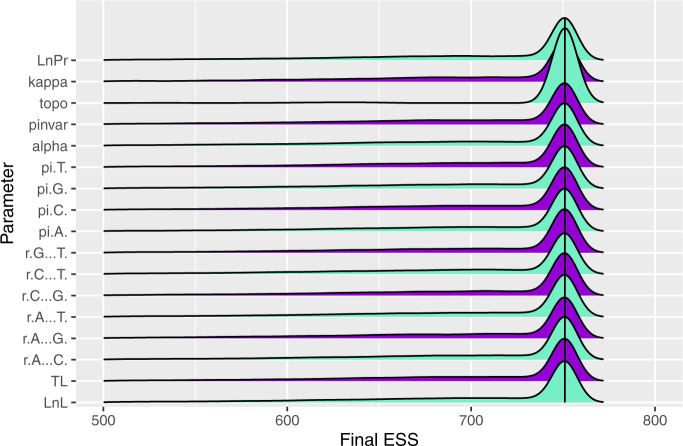
Density plot showing the frequency of ESS values for each parameter. Median values are denoted by solid vertical lines. The left tail extends to near zero but has been truncated for space.

A common intuition in phylogenetics is that effective sampling of the topology is more difficult than the other (continuously valued) parameters of the model. Counter to this intuition, we find that topology attained the highest final ESS on average (fig. 1) for runs of the same length. This seeming contradiction is explained by the larger number of tree-related MCMC proposals that MrBayes employs to counteract this difficulty, such that overall, the topology accumulates effective samples at similar or faster rates as the other parameters of the model.

**Fig. 2.**
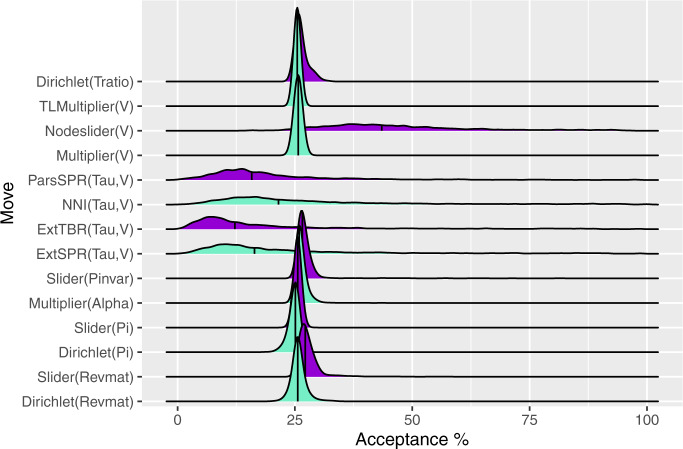
Density plots showing the frequency of acceptance rates for each move. Median values are denoted by solid vertical lines.

The remaining parameters do not show a consistent pattern for total ESS. Autocorrelation times, which are closely related to ESS, for all parameters were similar and slightly >1, likely due to the relatively sparse sampling that we used to make the handling of so many chains manageable (supplementary fig. S2, Supplementary Material online). Topology has the highest mean autocorrelation, although also the lowest median autocorrelation because the calculation of autocorrelation times for topology permits them to take only integer values, causing a mode at exactly 1.0 and another peak at exactly 2.0 but no values in between, whereas those for other parameters may take decimal values. Median acceptance ratios for MCMC moves were all in the targeted 20–70% range for one-dimensional proposals or 15–40% for multidimensional proposals, with most density centered around the typical target of 23% (fig. 2; Gelman et al. 1996; Yang 2006). This is expected given that MrBayes autotunes non-topology proposals to a default target of 25% acceptance. However, the variation around these medians is highly unequal. Moves that act on the tree topology, ExtSPR, ExtTBR, NNI, ParsSPR, and Nodeslider have the largest variation across analyses, and range from as low as 0.4% for ExtTBR up to 98.7% for ExtSPR. This indicates that on average, topology proposals achieve desirable acceptance rates, but that this can vary widely across analyses and appears to be data set dependent. As a discrete parameter, tree topology has an upper bound on move acceptance rates that is determined by the posterior, and this may partially drive the observed acceptance patterns (Peskun 1973; Yang 2014). Particularly low acceptance rates for topology moves may occur when only one or a few trees have high posterior probability, in which case, nearly all proposed changes to topology would be rejected. Conversely, when there is little information in the data to distinguish among topologies, large numbers of topology proposals may be accepted.

Based on these commonly used diagnostics, the majority of chains pass single-chain diagnostics (ESS and topological ESS), PSRF, and have high split frequency correlations, but less than half successfully achieve the targeted ASDSFs. Chains that did pass all diagnostics rapidly achieved high ESS within a few million generations post-burn-in (burn-in extends until generation 2,500). Autocorrelation times are low and move acceptance rates fall within targeted ranges on average. Analyses that do converge as indicated by all diagnostics do so rapidly and with targeted acceptance rates, suggesting that after filtering out poorly converged analyses, these commonly used MCMC settings allow for broadly satisfactory performance, which is reassuring if not unexpected. In preliminary analyses that we performed for PhyLoTA data sets that included more MCMC samples overall (not shown), we found that the majority of analyses did hit the targeted ASDSF threshold, suggesting that more of the analyses we present here might perform satisfactorily if more samples were collected by running longer chains.

### Congruence among Diagnostics

**Fig. 3.**
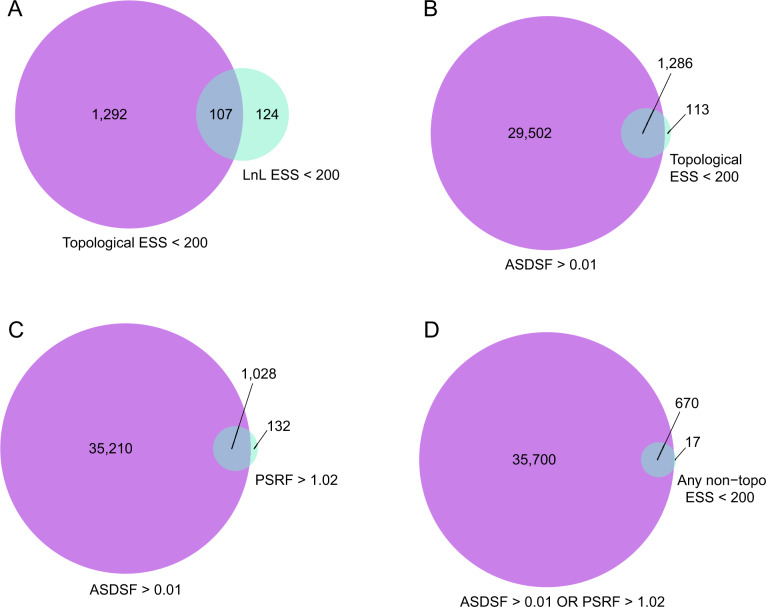
Venn diagram showing the number of chains that fail either one or both convergence diagnostics indicated under each circle. Part (*A*) shows chains that fail topological ESS, LnL ESS, or both. Part (*B*) shows chains that fail ASDSF, topological ESS, or both. Part (*C*) shows chains that fail ASDSF, PRSF, or both. Part (*D*) shows chains that fail ASDSF, any non-topological ESS, or both.

Among chains with detectably poor performance, we found a frequent lack of agreement among convergence diagnostics. There is not strong overlap among chains that have LnL ESS < 200 and chains that have topological ESS < 200 (fig. 3*A*). Chains that have a topological ESS < 200 tend to have at least one non-topology parameter fail to reach ESS of 200 with an elevated frequency but the majority of these still achieve high ESS for all non-topology parameters: 85% of analyses that fail topological ESS have ESS > 200 for all non-topology parameters. This shows that topological ESS is capturing unique aspects of the MCMC that are not detected by the LnL ESS, despite LnL broadly summarizing the fit of the model to the data. Topology is a unique parameter in that it is discrete and not easily summarized in Euclidean space. As a result, ESS for LnL and non-topology parameters may not easily detect poorly mixing topologies (Huelsenbeck et al. 2002; Nylander et al. 2008; Whidden and Matsen 2015; Lanfear et al. 2016), resulting in the observed incongruence.

We found that low topological ESS values were much more likely to have high ASDSF values than they were to fail other ESS values, with most, but not all, chains that fail topological ESS also failing ASDSF (fig. 3*B*). The comparatively small number of cases in which topological ESS detects failed convergence but ASDSF does not may indicate cases in which there is overall concordance among chains, but individual chains may exhibit high autocorrelation or infrequent, erratic changes. In contrast, many chains fail ASDSF that do not fail topological ESS, suggesting that most problems with topological convergence are not detectable by examining the within-chain behavior.

We also identified low congruence between ASDSF and the correlation of split frequencies between chains. This surprised us because both metrics measure the similarity of tree topologies among multiple chains in similar ways. ASDSF may potentially be more sensitive to poor mixing for a small number of splits, whereas the overall correlation of split frequencies may remain high if only a small number of splits occur at different frequencies among clades. This indicates that researchers should visually inspect plots of split frequencies, available in the RWTY package (Warren et al. 2017), or standard deviations of specific splits, as output by MrBayes when generating consensus trees, even when the overall correlation is high, in order to check for small numbers of outliers.

We identified chains that failed either ASDSF or PSRF, indicating a failure of independent chains to converge onto the same topologies or distributions of non-topology parameters, respectively. Many more chains failed ASDSF than PSRF, and most of the chains that failed PSRF also failed ASDSF (fig. 3*C*). Additionally, the vast majority of chains that failed PSRF or ASDSF achieved ESS values >200 (fig. 3*D*). This highlights the potential for individual chains to reach stationarity without evidence of convergence issues, even when independent chains are sampling from different regions of parameter or tree space (Huelsenbeck et al. 2002; Whidden and Matsen 2015).

Different diagnostics assess different properties of MCMC convergence, and any given diagnostic may not detect failed convergence in a range of scenarios. It is thus expected that some chains will fail some diagnostics but not others. Using all applicable convergence diagnostics is therefore necessary to have the greatest possible confidence that chains have sampled adequately. This includes using both single- and multichain diagnostics, as well as diagnostics that focus on both continuous parameters and topologies. In our anecdotal experience, it is common for researchers to focus almost exclusively on ESS and trace plots for continuous parameter values, without also considering statistics that focus specifically on topology. The inclusion of topology-specific diagnostics is crucial, given the primacy of topology in phylogenetic analysis and general concerns about the difficulty of sampling topologies as compared with more standard numerical parameters (Huelsenbeck et al. 2002; Nylander et al. 2008; Whidden and Matsen 2015; Lanfear et al. 2016). Furthermore, as we demonstrate here, failing to examine ASDSF may overlook the most common phylogenetic MCMC failures.

### Data Set Characteristics and MCMC Performance

We used multiple regressions to determine if ESS values for each parameter were correlated with the numbers of taxa or characters across data sets. We found that the number of taxa was a significant predictor of ESS values for nearly all parameters, but the number of characters was significant only for ESS of the *α* parameter of the gamma distribution modeling among-site rate heterogeneity, the proportion of invariable sites, and topology. Even then, these regression coefficients were considerably lower than those for number of taxa (table 2). Regression coefficients between numbers of taxa and parameter ESS values are negative (i.e., indicating worse convergence as the number of taxa increases) for all parameters except proportion of invariable sites, likely a result of the fact that the proportion of invariable sites should decrease as the number of taxa increases. The strongest negative correlations are between number of taxa and LnL ESS, ESS for several substitution rate parameters of the GTR model, and topological ESS.

**Table 2.** Regression Coefficients and *P* Values from Multiple Regressions Showing the Effect of Numbers of Taxa and Characters on ESS Values for Various Parameters.

	Taxa		Characters	
	Coefficient	*P*	Coefficient	*P*
LnL	−0.26	5.1 × 10^−101^	0.00	0.78
TL	−0.10	3.6 × 10^−17^	0.00	0.27
*q*_AC_	−0.05	2.2 × 10^−03^	0.00	0.41
*q*_AG_	−0.17	3.2 × 10^−26^	0.00	0.44
*q*_AT_	−0.14	2.7 × 10^−20^	0.00	0.22
*q*_CG_	−0.04	1.6 × 10^−02^	0.00	0.53
*q*_CT_	−0.16	2.8 × 10^−25^	0.00	0.40
*q*_GT_	−0.06	1.3 × 10^−4^	0.00	0.44
*π*_A_	−0.07	3.4 × 10^−7^	0.00	0.84
*π*_C_	−0.10	3.2 × 10^−14^	0.00	0.06
*π*_G_	−0.10	5.2 × 10^−15^	0.00	0.81
*π*_T_	−0.08	2.6 × 10^−9^	0.00	0.61
*α*	0.00	0.87	0.00	1.6 × 10^−3^
I	0.05	3.0 × 10^−3^	0.00	4.8 × 10^−3^
Topology	−0.16	1.6 × 10^−113^	−0.01	2.3 × 10^−88^
*κ*	−0.02	0.27	0.00	0.38
LnPr	−0.06	2.3 × 10^−03^	0.00	0.93

The negative associations between the number of taxa in an analysis and ESS values, particularly LnL ESS and topological ESS, suggest that increasing the number of taxa can lead to considerable challenges with MCMC performance. This is expected because the size of tree space rapidly becomes enormous for even modest numbers of taxa (Felsenstein 2004) and exploring that tree space efficiently is one of the key challenges for phylogenetic inference. By contrast, there is very little correlation between any ESS values and number of characters in the analyses. We suspect that this is driven by the fact that increasing the amount of characters in an alignment may simply add congruent signal, leading to a more peaked stationary distribution that is easier to sample from, or add heterogeneity and conflict, which can lead to islands in the stationary distribution and making mixing more difficult. In addition, increasing the number of characters may simply add redundant phylogenetic signal that has little impact on the analysis (Lewis et al. 2016). Therefore, the effect of the number of characters on MCMC performance will depend on the specific data set. The impact of the number of characters may increase for large, genome-scale data sets. However, as data sets grow to much larger scales, data are typically modeled under more complex models including multiple data partitions, which are likely to have further effects on convergence that are beyond the scope of this study.

**Fig. 4.**
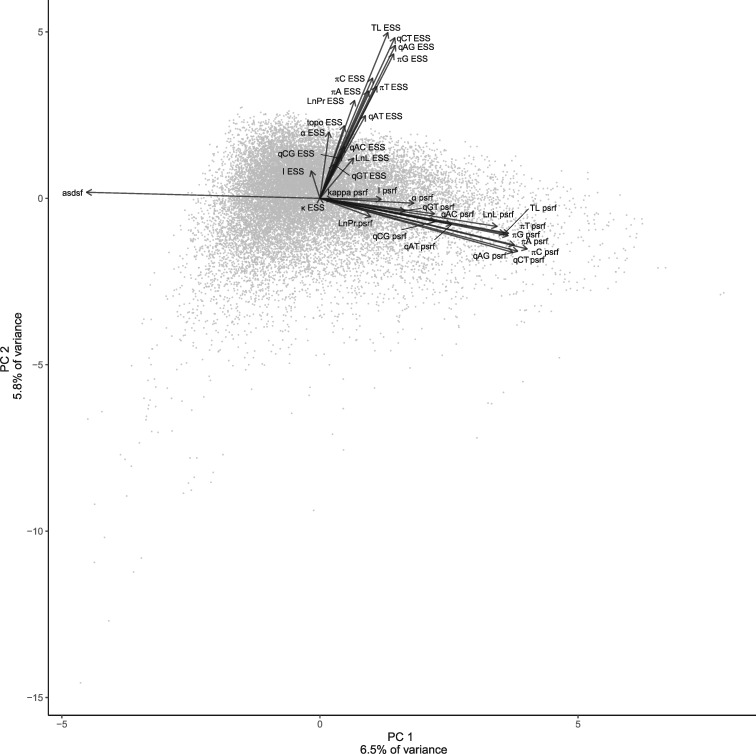
PCA of core convergence diagnostics showing loadings of variables. Loadings have been rescaled to be visible on the same scale as the PCs.

Principal components analysis (PCA) of core convergence diagnostics shows the relationships among ESS values for all parameters, topological ESS, ASDSF, and PSRF for all parameters. We find that PC1 explains 6.5% of variance with ASDSF strongly loading negatively and PSRF for multiple parameters loading most positively, indicating a prime role for multichain diagnostics in separating out the performance of data sets (fig. 4). Both ASDSF and PSRF indicate better convergence with decreasing values, and so the opposing loadings of these two diagnostics indicates a separation between data sets that converge comparatively poorly for topology (ASDSF) or non-topology parameters (PSRF). PC2 explains 5.8% of variance and largely positively loads ESS values (TL ESS most strongly) and negatively loads PSRF values, with high values of PC2 representing high ESS and low PSRF, that is, good convergence, exclusive of the influence of ASDSF, which loads minimally on PC2. The minimal loading of ASDSF and only slightly higher loading of PSRF suggests that PC2 reflects primarily within-chain mixing, whereas PC1 largely describes how multiple chains converging onto the same distributions, and that these two aspects of convergence are largely independent.

Using multiple regression, we show that the number of taxa, number of characters, and TL/branch (the average branch length over a tree) are all highly significant predictors of PCs 1 and 2, with TL/branch exhibiting the highest regression coefficient by orders of magnitude (table 3). The overall adjusted *R*^2^ are low, although we expect this given the number of idiosyncratic factors that influence MCMC behavior for any given chain. Short branches are one of the hallmarks of difficult phylogenetic problems (e.g., Jarvis et al. 2014; Leaché et al. 2016; Burbrink et al. 2020), and so it makes sense that ASDSF decreases as TL/branch increases.

**Table 3.** Regression Coefficients and *P* Values from Multiple Regressions Showing the Effect of Numbers of Taxa, Number of Characters, and TL/Branch on Principal Components of Convergence Diagnostics (first two rows) and Acceptance Rates (third row).

	# Taxa		# Characters		TL/Branch		Adjusted *R*^2^
	Coefficient	*P* Value	Coefficient	*P* Value	Coefficient	*P* Value	
PC1 of diagnostics	−7.7 × 10^−03^	1.0 × 10^−237^	−7.3 × 10^−05^	3.3 × 10^−17^	6.1	7.9 × 10^−204^	1.6 × 10^−1^
PC2 of diagnostics	−4.3 × 10^−03^	4.3 × 10^−75^	4.9 × 10^−05^	2.4 × 10^−08^	−3.6	9.3 × 10^−72^	2.1 × 10^−2^
PC1 of acceptances	−4.5 × 10^−03^	8.4 × 10^−23^	−1.4 × 10^−03^	0.00	−31.2	0.00	0.39

**Fig. 5.**
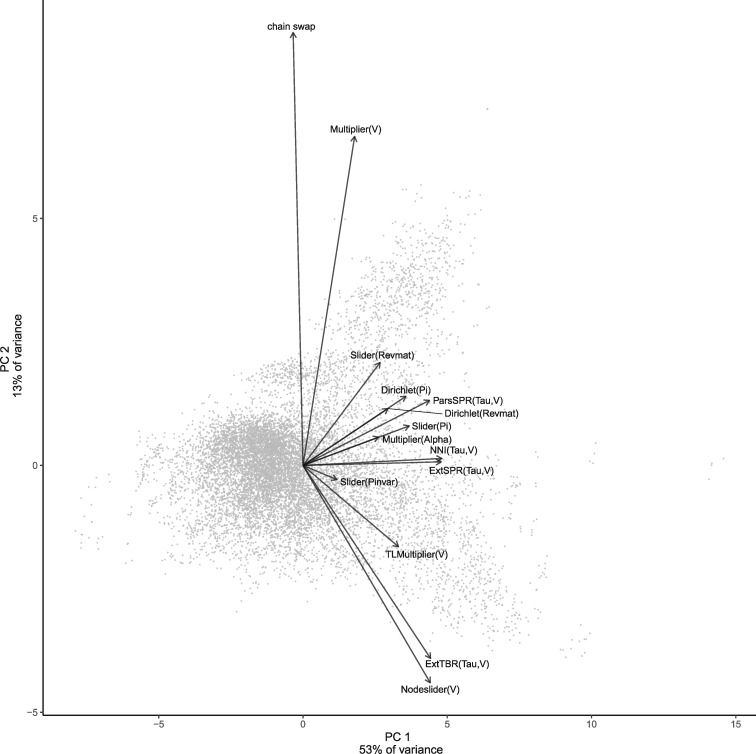
PCA of acceptance rates showing loadings of variables. Loadings have been rescaled to be visible on the same scale as the PCs.

In our PCA of acceptance rates, PC1 explains 53% of variation, with topology proposals loading most strongly and chain swaps loading slightly negatively (fig. 5). Number of taxa, number of characters, and TL/branch are all significant predictors of acceptance PC1 in a multiple regression, with TL/branch having a much larger absolute correlation coefficient than number of taxa or characters (table 3). In this case, the corrected *R*^2^ for the multiple regression is relatively high at 0.39, indicating that numbers of taxa and characters and particularly TL/branch are major predictors of the ability of phylogenetic MCMC to mix effectively. These effects may be better reflected in the acceptance rates than in the diagnostics partially because we performed analyses on sets of chains that have met all convergence diagnostic thresholds, perhaps limiting the variation in these end result diagnostics, whereas more variation is present in acceptance rates. We also expect that this is a real pattern, as the acceptance rates directly reflect the movements of the MCMC through parameter space, rather than the end result distributions. That is, even when convergence is harder to achieve and distributions are more rugged, with enough samples and thinning, good convergence as evidenced by ESS, ASDSF, etc., should still be achievable, but chains may sample the parameter space less effectively. Additionally, higher acceptance rates do not necessarily correspond to improved convergence. TL/branch has a positive correlation with PC1 of diagnostics but negative correlation with PC1 of acceptance rates, indicating that as average branch length goes up, ASDSF and topology acceptance rates both go down. This can intuitively be explained as a result of more focused tree posteriors: When branches are long, there is often unambiguous support for a small number of trees, leading to low ASDSF but also low acceptance rates because most tree proposals will be rejected once the few most probable trees have been found.

**Fig. 6.**
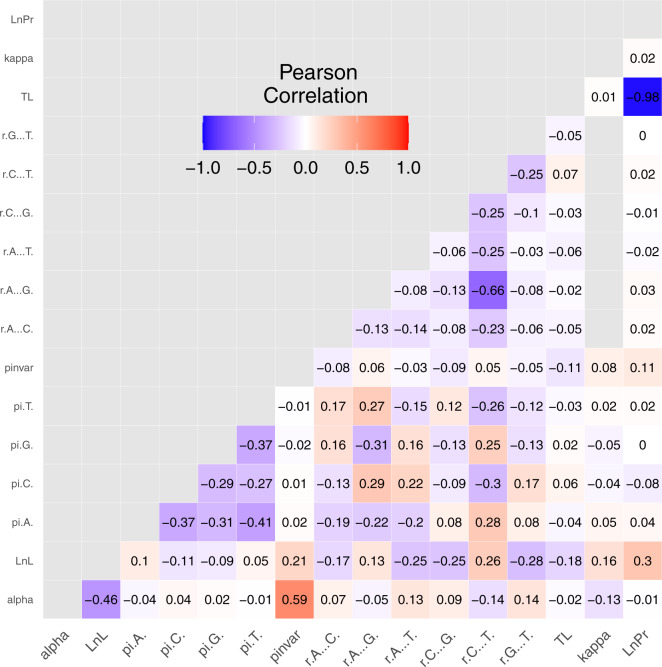
Correlation matrix showing the mean of Pearson correlation coefficients for values of pairs of parameters across individual chains.

To determine how parameters interact with each other in a chain, we calculated the average correlations among all parameters. We found a very strong negative correlation between LnPr and TL of −0.98 (fig. 6). This correlation is expected, because the tree length prior in MrBayes used for most analyses here is a Γ distribution on total tree length with branch lengths partitioned according to a Dirichlet distribution. The Γ distribution places more prior weight on relatively short trees than long trees (Rannala et al. 2012) and so drives this correlation. The amniotes data sets used an exponential prior on tree length, which also places higher prior weight on shorter trees giving the same expectation. A strong positive correlation with a median of 0.59 exists between *α*, the shape parameter for Γ-distributed substitution rate heterogeneity, and I, the proportion of invariable sites in the alignment. However, high correlations between these parameters do not seem to strongly affect MCMC sampling for either parameter, at least as measured by ESS. As the correlation between parameters of the I + Γ model has been the subject of considerable discussion (Sullivan et al. 1999; Mayrose et al. 2005; Yang 2006), we paid special attention to these parameters, and explored the effect of using I + Γ versus Γ in further detail.

### Improving Performance

#### Models with I + Γ versus Γ

We reanalyzed data sets for which the selected substitution model included I + Γ under a simpler model that included only Γ. Of the analyses that originally had poor convergence of I or Γ, these analyses failed a median of 5 fewer diagnostic thresholds (summing the number of failed diagnostics across chains within each analysis). Analyses that had already passed all diagnostic thresholds when implementing the I + Γ model did not perform worse when switching to a Γ-only model and showed zero median change in the number of failed diagnostics. Simplifying models from I + Γ to Γ tended to result in very small changes to tree topologies as measured by normalized Robinson–Foulds (RF) distances between original and reanalyzed chains when considering 95% or 50% consensus trees (supplementary fig. S3*a*, Supplementary Material online). Median RF distance increased when maximum clade credibility (MCC) trees were compared, as was expected due to the potential inclusion of poorly supported clades in such trees that are liable to change among different analyses.

Although the *α* parameter for the Γ-distributed rate heterogeneity is generally correlated with the proportion of invariable sites when the I + Γ model was used, this does not typically seem to result in problematic inference. Using the Γ rather than the I + Γ model can improve performance when MCMC performance is unsatisfactory, but most analyses pass all diagnostic thresholds even when using the I + Γ model. Tree topologies also change minimally with the use of Γ rather than I + Γ. These results together suggest that, when the tree topology is of primary interest, the I + Γ model is not generally as problematic as some (including ourselves) have worried (Sullivan et al. 1999; Mayrose et al. 2005; Yang 2006), but also that using Γ alone is sufficient to achieve essentially the same topologies as are inferred when using I + Γ. This coupled with the improved performance when switching from I + Γ to Γ for those analyses that do initially have low ESS suggests that there is little benefit to including both together, and a low risk that doing so will lead to higher incidence of performance issues. We therefore echo previous researchers and recommend that researchers select Γ rather than I + Γ as a typical starting point.

#### Heating and Model Averaging

Heating changes resulted in failing a median of one fewer diagnostic threshold (mean = 0.95). This is predominately a result of more chains passing ASDSF (table 4). The use of model averaging over substitution models (nst = mixed in MrBayes) provided less improvement to convergence, with a median change in the number of failed diagnostics of zero and a mean of 0.42. Again, the predominate change is an increase in the number of analyses that achieve satisfactory ASDSF (table 4). Using updated heating or model-averaged substitution models yielded trees that had low RF distances from the original trees for 95% and 50% consensus trees, but higher RF distances when comparing MCC trees (supplementary fig. S3*b* and *c*, Supplementary Material online). Given that heating does not change the model, there should be no change in tree topologies unless previous analyses had become stuck in local optima. These strategies both demonstrate the potential to improve convergence but are clearly not universal cures. Both strategies are useful to consider when attempting to fix convergence issues, but phylogeneticists will need to focus on diagnosing the specific causes of poor performance in their analyses and apply strategies tailored to these causes.

**Table 4.** Proportion of a Subset of Chains That Pass Diagnostic Thresholds before and after Altering Heating or Averaging over Substitution Models Using the Nst = Mixed Option in MrBayes.

	Pass ESS	Pass Topo ESS	Pass ASDSF	Pass SF Corr	Pass PSRF
Original	0.92	0.79	0.08	1.00	0.91
Nst = mixed	0.90	0.83	0.15	0.98	0.89
New heat	0.94	0.92	0.16	0.99	0.89

#### Manual Diagnosis and Adjustment

For the subset of analyses that we manually examined to determine causes of convergence failures, we found that many analyses were erratically sampling values for substitution and base frequency parameters of the GTR model. This usually indicated a substitution model that was too complex relative to the variation present in the data. Typically, using a simpler substitution model (HKY in this study) or empirical base frequencies improved performance considerably for continuous parameters. Analyses both failed fewer overall diagnostic thresholds and had high ESS for more parameters. However, ASDSF values remained low for most of these analyses (supplementary tables S2–S4, Supplementary Material online). When using the HKY rather than GTR model, RF distances for both 95% and 50% consensus trees were slightly lower than when using GTR models with empirical base frequencies, although we note that the sample size is low (supplementary fig. S4*a* vs. S4*b*, Supplementary Material online). RF distances are generally low when replacing the GTR model with HKY in these analyses, with the exception of MCC trees, as in other comparisons above. Unexpectedly, averaging over substitution models using the Nst = mixed option did not yield gains similar to switching to an HKY model or empirical base frequencies. We suspect that this may reflect a lack of sufficient data to guide the reversible-jump model toward a simpler, HKY-like model in these data sets. As expected, running analyses longer also improved performance and with low RF values as there is no change to the model, simply more samples from the posterior. Analyses failed fewer diagnostic tests and achieved ESS above 200 for many more parameters in these cases, and notably, three out of the four analyses that we ran longer without changing any further parameters achieved desirable ASDSF values. Many chains, especially for more complex analyses that feature more taxa, characters, or model parameters than we have considered here, will require many more than 10M generations to achieve adequate convergence (Whidden and Matsen 2015). The most common problems in this set of analyses therefore seems to be data sets that may have had low information and been overparameterized with more complex substitution models, analyses that were larger and more complex, requiring additional generations to adequately converge, or both.

## Conclusions

This survey of MCMC performance yields several lessons that are broadly instructive for users of Bayesian phylogenetic methods. The first is the importance of using all available convergence diagnostics. We find that different diagnostics detect different failures. The starkest differences are between topology and non-topology diagnostics; and between single- and multichain diagnostics. Given that topology is often the most important target of inference, and that diagnostics focusing on the continuous parameters in the model routinely fail to detect MCMC performance problems with topology, researchers should be motivated to apply topology-specific diagnostics to their phylogenetic MCMC analyses. Additionally, single-chain diagnostics cannot identify failures of independent chains to converge onto the same stationary distribution, and many chains that pass single-chain diagnostics fail multichain diagnostics. We reiterate the common recommendation in the field to always run several independent analyses and assess congruence between them.

We find that when MCMC performs poorly, adjusting heating of Metropolis-coupled chains using coarse rules, or utilizing reversible-jump model averaging over substitution models can provide improved convergence, but do not alone surmount the problem in a majority of cases. Instead, hunting down the culprits of failed MCMC will often require case by case examination to identify the specific causes, which are likely to be idiosyncratic. Because of this, additional computational tools that help automate this time intensive task would be a major benefit for the field. Although we have focused on cases where MCMC has failed and how to identify and rectify it, we would like to close by pointing out that the pursuit of MCMC convergence does not appear to be particularly bleak, at least for the simple and commonly employed Bayesian phylogenetic analyses that we examine here. For larger data sets containing multiple gene regions, or mixtures of data types, MCMC performance issues are expected to be more pervasive and difficult to solve. Similarly, these issues become more pronounced as inference models become more complex and hierarchical, as is the case for joint inference of divergence times and phylogeny or inference of species trees under the multispecies coalescent model. A large set of related topics might be explored using the data set we have assembled here, or similar data sets derived from the literature. These areas include exploring the performance of diagnostics that see wide use in Bayesian statistics generally, but little use in phylogenetics (e.g., Geweke’s test, the Heidelberger and Welch diagnostic); other basic techniques for assessing MCMC performance generally (e.g., comparing the divergence between the prior and posterior distributions); focusing in more detail on behaviors of Metropolis-coupled chains (e.g., Brown and Thomson 2018); and additional strategies for optimizing MCMC performance, such as further optimizing acceptance rates, including using guided and adaptive tree proposals (Höhna and Drummond 2012; Meyer 2019; Zhang et al. 2020). As analyses become more complex, more complex strategies will be needed to detect, diagnose, and resolve the difficulties.

## Materials and Methods

We assembled MrBayes v3.2.2 and 3.2.5 (Huelsenbeck and Ronquist 2001; Ronquist et al. 2012) MCMC output from three previous studies (Barley and Thomson 2016; Brown and Thomson 2017; Richards et al. 2018) and conducted a new set of 10,382 MrBayes v3.2.5 analyses using data sets assembled from the PhyLoTA database (Sanderson et al. 2008), for a total of 18,588 analyses that span many clades across the tree of life and widely different time scales. These previous studies carried out analyses of a large number of alignments in order to investigate information content across the amniote phylogeny (Brown and Thomson 2017), how model fit impacts DNA barcoding efforts among closely related taxa (Barley and Thomson 2016), and the extent of systematic error in analyses of mitochondrial genomic data within the major lineages of amniotes (Richards et al. 2018).

MrBayes 3.2.2 was used in analyses from Barley and Thomson (2016) and Brown and Thomson (2017) and MrBayes 3.2.5 was used in Richards et al. (2018). The default branch length prior for MrBayes in v3.2.2 was an exponential prior, and this was used for analyses in Brown and Thomson (2017), whereas a compound Dirichlet prior was used in analyses from Barley and Thomson (2016) and Richards et al. (2018) (Yang 2007; Rannala et al. 2012). This updated branch length prior was developed to combat issues of pathologically long trees sometimes being recovered when the exponential prior was used (Brown et al. 2010; Marshall 2010). We note that this problem is more common in partitioned analyses (Brown et al. 2010), and so we do not expect this to be common issue in the data sets from Brown and Thomson (2017) as these and the data sets that we collected from the two other studies are all unpartitioned, single-gene analyses. We used default topology proposals, which were the same in both versions of MrBayes: extended tree bisection regrafting (abbreviated ExtTBR in MrBayes), extended subtree pruning regrafting (SPR; abbreviated ExtSPR in MrBayes), parsimony-biased SPR (abbreviated ParsSPR in MrBayes), and nearest neighbor interchange (NNI in MrBayes and elsewhere). All analyses from previous studies executed two independent runs of four Metropolis-coupled chains. In each study, best fit substitution models for each data set were identified using external software. Barley and Thomson (2016) additionally informed compound Dirichlet branch lengths priors based on maximum-likelihood estimates, including and adjusted heating of Metropolis-coupled chains for some analyses to improve convergence.

For each study, we gathered the Nexus input file used to run the analysis, the MrBayes .t and .p files containing the tree and parameter samples recorded through the Markov chain for both of the independent runs, the .log files (not available for Brown and Thomson 2017), and other associated output files. For the new MrBayes analyses conducted here, we analyzed all data sets from the PhyLoTA database that contained between 25 and 250 taxa. We downloaded data sets, aligned the data using Muscle (Edgar 2004), trimmed ragged ends containing more than 75% missing sites using the trimEnds function of the R package ips (Heibl 2019: https://cran.r-project.org/web/packages/ips/), chose a substitution model for each alignment using the AICc criterion in JModelTest selecting only among models that are implemented in MrBayes (Posada 2008), generated MrBayes input files, and ran all analyses in MrBayes on the University of Hawaii HPC cluster using a set of custom scripts. We selected models of substitution using JModelTest in order to replicate what is perhaps the most common historical choice for model selection. We ran four independent Metropolis-coupled (one cold chain and three heated chains) MCMC runs for 10 million generations and thinned the chains to retain 1,000 MCMC samples for further analysis. The scripts and further detail about the settings used in this pipeline are available in the github repository associated with this study (https://github.com/seanharrington256/MCMC_convergence). We also pruned samples from the chains that arose from previous studies so that they match the analyses from the PhyLoTA database. To do so, we removed samples after 10 million generations and thinned the remaining samples to retain 1,000 samples. This was done so that chains could be directly compared among the studies. Here, we are less interested in ensuring that all chains have converged than in the differences among convergence properties for different data sets under similar analytical conditions.

The MCMC chains we have included here are broadly similar in that they were all run with MrBayes (differing only by minor version), all utilize Metropolis coupling, are single-gene (or potentially genes and associated flanking sequences in the case of the PhyLoTA analyses) with varying numbers of taxa and characters (supplementary fig. S5, Supplementary Material online), are unpartitioned, and use predominately default priors, with few differences in priors (e.g., substitution models vary, branch length priors differ between analyses between Brown and Thomson [2017] and other studies). We chose this set of analyses due to a combination of availability (full output files from MCMC chains are almost never reported), comparability among chains, and computational considerations for the amount of disk space, processing power, and time needed to store and analyze the chains. Although many studies now include genome-scale data sets and more complex models, understanding how MCMC chains behave empirically in these relatively simple cases is an important preliminary step. We expect the lessons learned here to translate to more complex data sets with the caveat that interactions among properties of the data sets and chains will only become more complex as larger data sets and more complex models such as molecular clocks and partition models are included.

We used custom R v3.3.2 (R Core Team 2016) scripts to calculate an array of convergence diagnostics along with several features of the data and analyses from each set of MrBayes output, then summarized broad patterns in these diagnostics across all data sets. These 16 total diagnostics and features are listed in supplementary table S1, Supplementary Material online. In general, we calculated ESS for each parameter in the model, as well as the log likelihood (LnL), log prior (LnPr). We recognize that these last measures are not parameters in the model, although we occasionally refer to them as such for convenience when discussing, for example, trends in ESS across parameters sampled in the MCMC. A fixed 25% burn-in was removed before calculating all diagnostics. Diagnostics were calculated using functions from or modified from the RWTY (Warren et al. 2017), CODA (Plummer et al. 2006), tracerer (Bilderbeek and Etienne 2018), and BONSAI (https://github.com/mikeryanmay/bonsai, last accessed February 2018) packages, or were extracted directly from the MrBayes output.

We investigated the effects of data set properties (including numbers of taxa and characters) on convergence diagnostics using multiple regressions. We also used PCA (using the pcaMethods package in R: Stacklies et al. (2007) to gain a more complete picture of the correlations among convergence diagnostics. We performed one PCA on what we refer to as “core diagnostics”: ESS values for all parameters, topological ESS, ASDSF, and PSRF for all parameters. We performed a second PCA on acceptance rates of MCMC moves and the acceptance rate of chain swaps between the cold chain and first heated chain. We performed this second PCA excluding the amniotes data set of Brown and Thomson (2017) as we did not have access to files necessary to extract acceptance information for these analyses. Input data were centered and scaled to unit variance prior to PCA. We used multiple regression to determine the effect of number of characters, number of taxa, and average branch length across each tree (tree length per branch, TL/branch) on the first and second principal components of our core diagnostics PCA as metrics and separately on PC1 of the PCA on acceptance rates.

We used correlations to examine the relationships among parameters through chains, paying particular attention to the correlation between the proportion of invariable sites and the *α* parameter for Γ distributed among-site rate heterogeneity in models that contained both parameters (I + Γ models) due to the concerns surrounding the potential interaction of these parameters discussed in the introduction. All correlations among parameters were calculated as Pearson correlations. All relationships among MCMC properties and data set characteristics were investigated only on chains that passed all relevant convergence diagnostics.

After summarizing convergence diagnostics across these data sets, we identified a subset of analyses with potentially poor MCMC performance (*n* = 1,979) and binned them according to the following criteria: 1) ESS < 200 for at least one continuous parameter, 2) topological ESS (as approximate topological ESS [Lanfear et al. 2016]) < 200 and LnL ESS < 200, 3) topological ESS < 200 but LnL ESS > 200, and/or 4) ASDSF > 0.01. To attempt to determine if broad-scale recommendations could be made to improve performance of these analyses, we implemented two sets of reanalyses. Metropolis coupling can be useful in improving the exploration of parameter space (Geyer 1991; Altekar et al. 2004), and so we first altered the heating of each analysis to determine if this could result in improved performance across the selected analyses. We used the acceptance rates of the proposed swaps between the cold and heated chains to guide heating changes by first coarsely dividing poorly performing analyses into high (>0.5) and low (<0.5) acceptance rate categories. We doubled the heating for the high category, as high acceptance rates indicate that chains may be trapped in similar areas of parameter space and that these analyses may benefit from bolder proposals. We halved the heating for the low acceptance category, as low acceptance indicates that the heated chains may be spending too much time in low-probability areas of parameter space leading to inefficient mixing. Second, we changed the substitution models for the same set that we altered heating for to implement model averaging over all subsets of the GTR model (retaining the original modeling of invariable and/or Γ distributed site rates) by using the reversible-jump nst = mixed option in MrBayes (Huelsenbeck et al. 2004). Upon reanalyzing the data sets using each of these strategies, we compared convergence diagnostics of the original and reanalyzed output. We also compared RF tree distances (Robinson and Foulds 1981) among the original and reanalyzed chains to check if these changes affected estimates of topology.

We also selected a set of analyses (*n* = 1,060) that had originally been analyzed using a model that incorporated both invariable sites and Γ-distributed rates (I + Γ) to reanalyze using only Γ, as this can account for extremely low-rate sites while avoiding potential interaction among the two parameters. As above, we compared convergence diagnostics and tree topologies of the original analyses including I + Γ to those of the analyses including Γ only.

Finally, we sought to reanalyze a smaller set of poorly performing analyses individually. We selected 20 analyses that had ESS values below 200 for large numbers of parameters. We then manually examined trace plots and convergence diagnostics for each of these analyses individually to try to determine the specific cause of the problem. We reanalyzed each of the data sets using a strategy targeted toward what seemed to be the most probable culprit, including using simpler substitution models or simply running chains longer. Following these reanalyses, we compared the convergence diagnostics and topology to the original chains.

## Supplementary Material 

Supplementary data are available at *Molecular Biology and Evolution* online.

## Supplementary Material

msaa295_Supplementary_Data
