# Supplementary material for: Properties of Markov Chain Monte Carlo Performance across Many Empirical Alignments
Source: Mol Biol Evol. 2020 Nov 13;38(4):1627–40. doi: 10.1093/molbev/msaa295 (PMC8042746; doi:10.1093/molbev/msaa295)
Supplement: msaa295_Supplementary_Data [file msaa295_supplementary_data.pdf]

Figure S1. Density plot showing time in generations taken for each parameter to reach an ESS value of 200. ESS values were calculated every 1M generations rather than at every sampled interval for computational tractability. Median values are denoted by solid vertical lines. The right tail extends to 10 million but remains flat has been truncated for space.

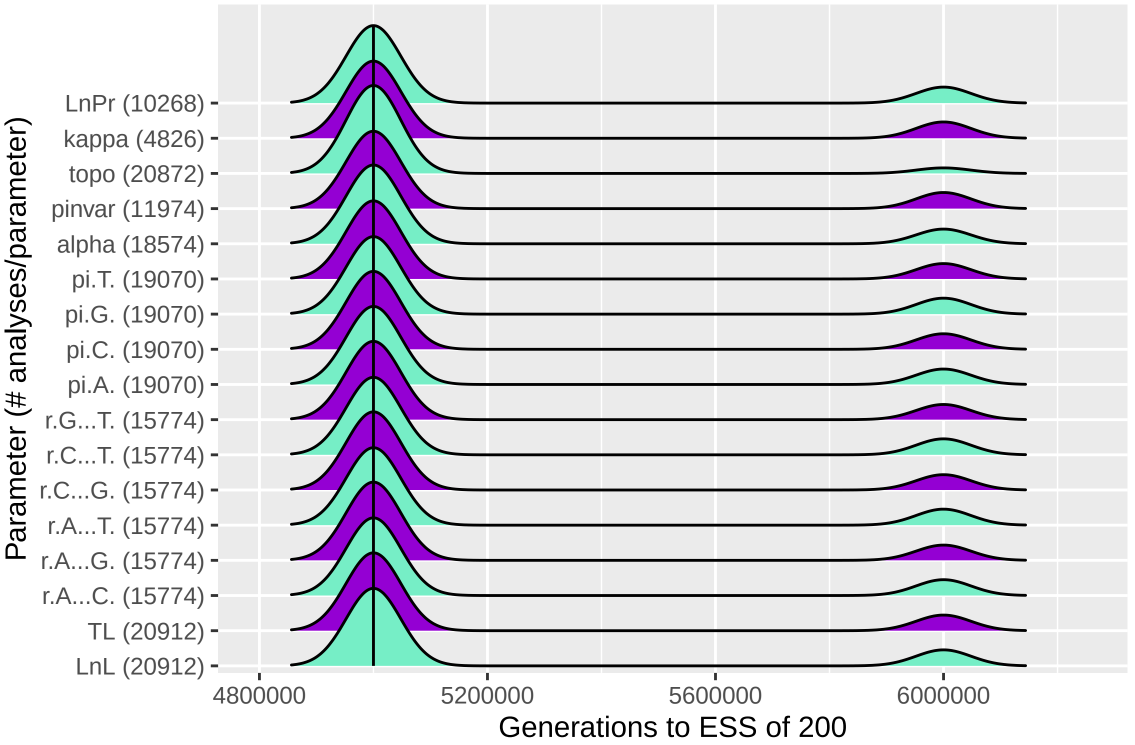

LnPr (10268)
kappa (4826)
topo (20872)
pinvar (11974)
alpha (18574)
pi.T. (19070)
pi.G. (19070)
pi.C. (19070)
pi.A. (19070)
r.G...T. (15774)
r.C...T. (15774)
r.C...G. (15774)
r.A...T. (15774)
r.A...G. (15774)
r.A...C. (15774)
TL (20912)
LnL (20912)
Parameter (# analyses/parameter)
4800000
5200000
5600000
6000000
Generations to ESS of 200

Figure S2. Density plot showing the frequency of autocorrelation times for each parameter. Median values are denoted by solid vertical lines. The right tail extends past 350 but has been truncated for clarity.

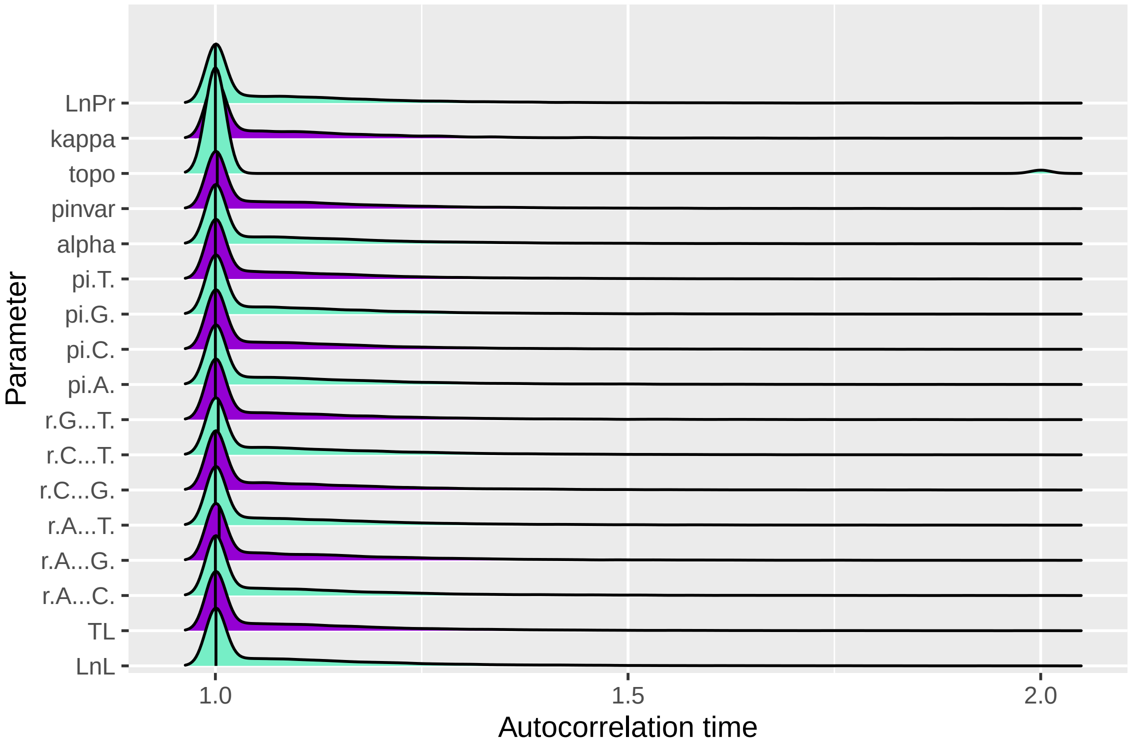
LnPr
kappa
topo
pinvar
alpha
pi.T.
pi.G.
pi.C.
pi.A.
r.G...T.
r.C...T.
r.C...G.
r.A...T.
r.A...G.
r.A...C.
TL
LnL
Parameter
1.0
1.5
2.0
Autocorrelation time

Figure S3. Histograms of the normalized Robin-Foulds (RF) differences among original and reanalyzed sets of chains. A) The top row contains chains originally analyzed using I+Γ and reanalyzed using Γ only. B) The middle row contains chains reanalyzed using the nst=mixed option for reversible-jump model averaging over substitution models. C) The bottom row contains chains reanalyzed using altered heating. The left column shows normalized RF distances between 95% consensus trees. The middle column shows normalized RF distances between 50% consensus trees. The right column shows normalized RF distances between maximum clade credibility trees.

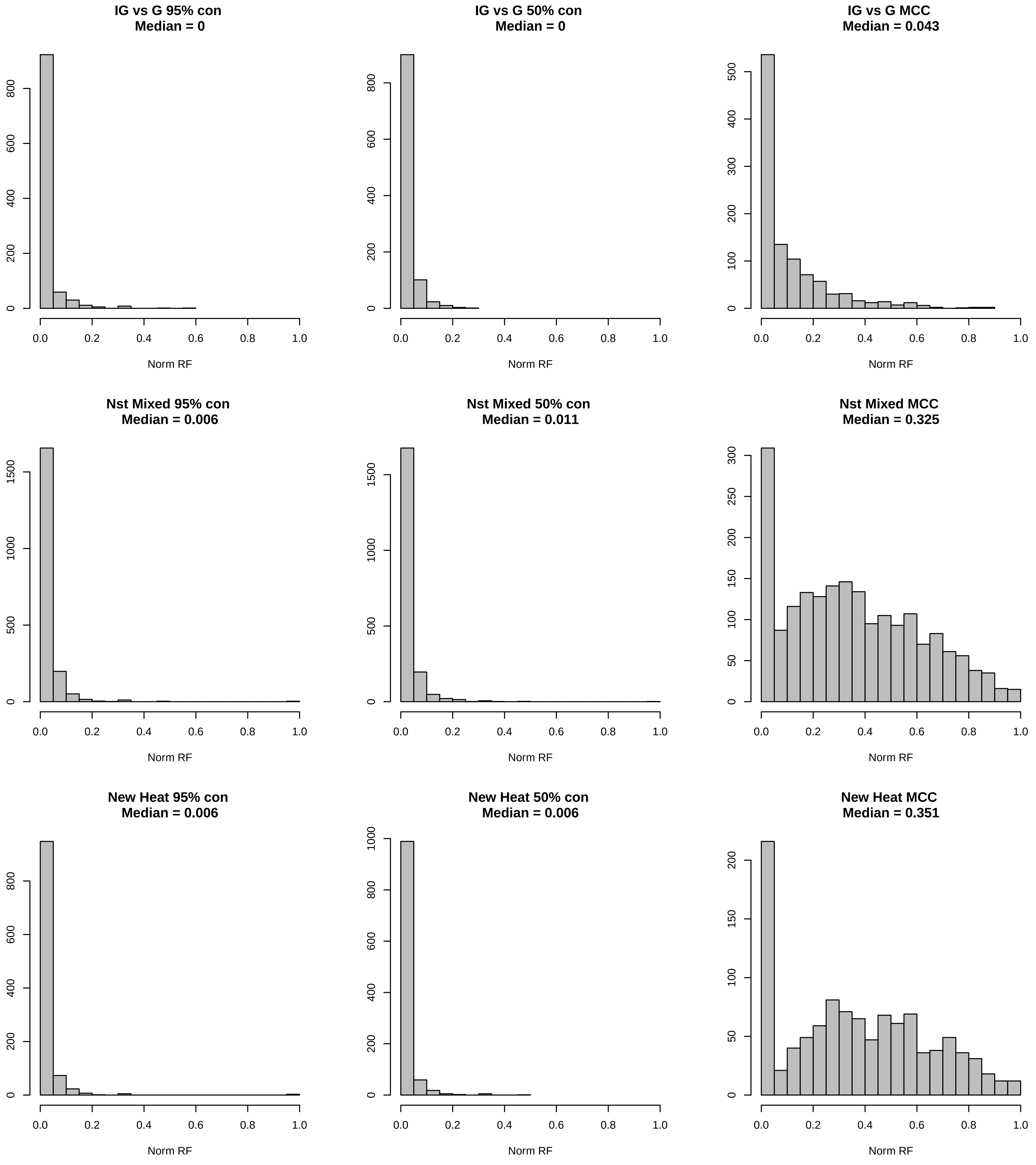
IG vs G 95% con
Median = 0
IG vs G 50% con
Median = 0
IG vs G MCC
Median = 0.043
Nst Mixed 95% con
Median = 0.006
Nst Mixed 50% con
Median = 0.011
Nst Mixed MCC
Median = 0.325
New Heat 95% con
Median = 0.006
New Heat 50% con
Median = 0.006
New Heat MCC
Median = 0.351
Norm RF

Figure S4. Histograms of the normalized RF distances among original and manually reanalyzed chains. A) The top row contains chains reanalyzed using the HKY model. B) The second row from the top contains the chains reanalyzed using empirical rather than estimated base frequencies. C) The third row from the top contains chains that were run for 30 M rather than 10 M generations while still retaining a total of 1,000 samples. D) The bottom row contains chains that were run for 30 M generations and retaining a total 3,000 samples. The left column shows normalized RF distances between 95% consensus trees. The middle column shows normalized RF distances between 50% consensus trees. The right column shows normalized RF distances between maximum clade credibility trees.

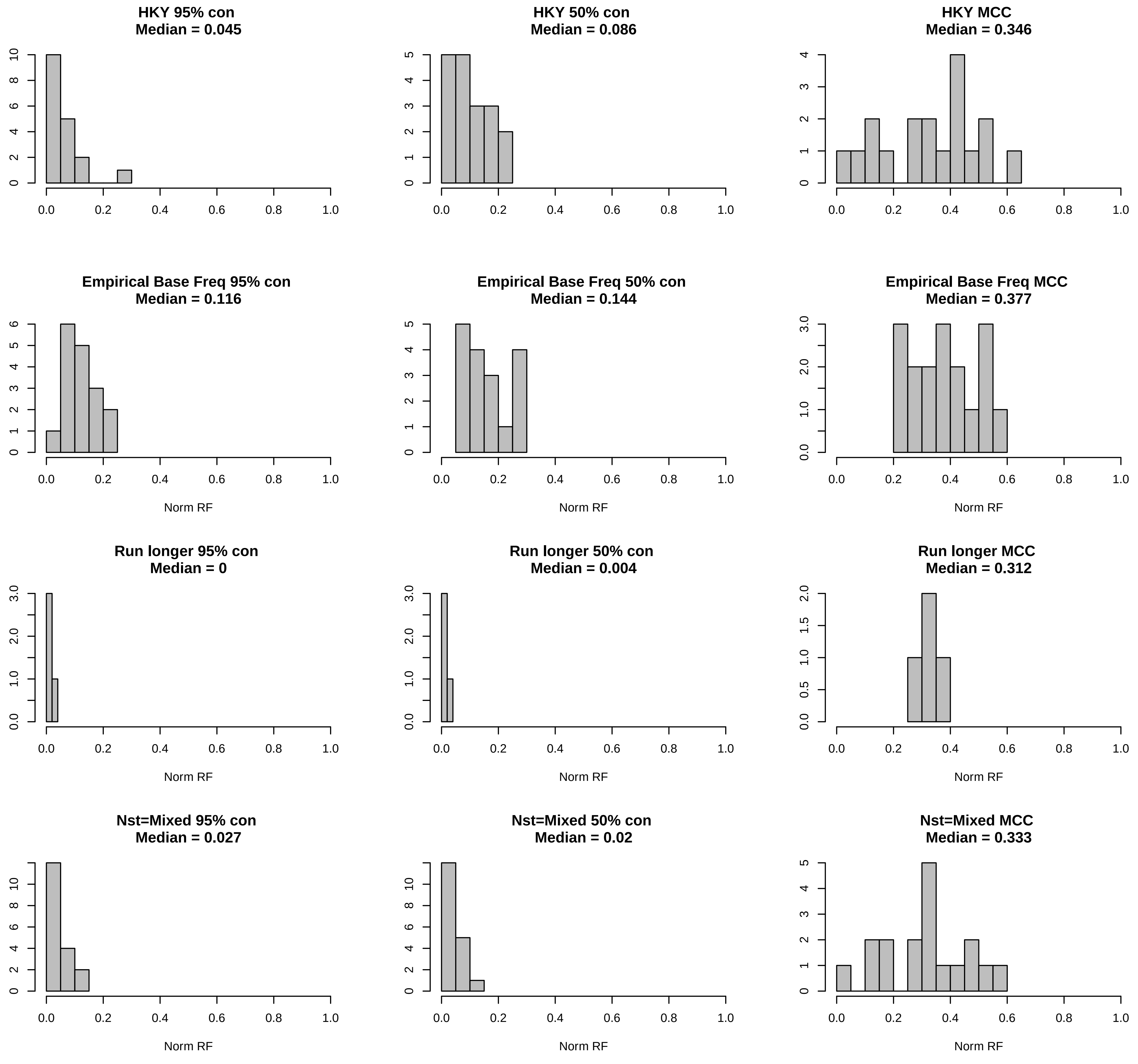
HKY 95% con
Median = 0.045
HKY 50% con
Median = 0.086
HKY MCC
Median = 0.346
Empirical Base Freq 95% con
Median = 0.116
Empirical Base Freq 50% con
Median = 0.144
Empirical Base Freq MCC
Median = 0.377
Run longer 95% con
Median = 0
Run longer 50% con
Median = 0.004
Run longer MCC
Median = 0.312
Nst=Mixed 95% con
Median = 0.027
Nst=Mixed 50% con
Median = 0.02
Nst=Mixed MCC
Median = 0.333
Norm RF

Figure S5. Numbers of characters and taxa included in each of the datasets analyzed in this study.

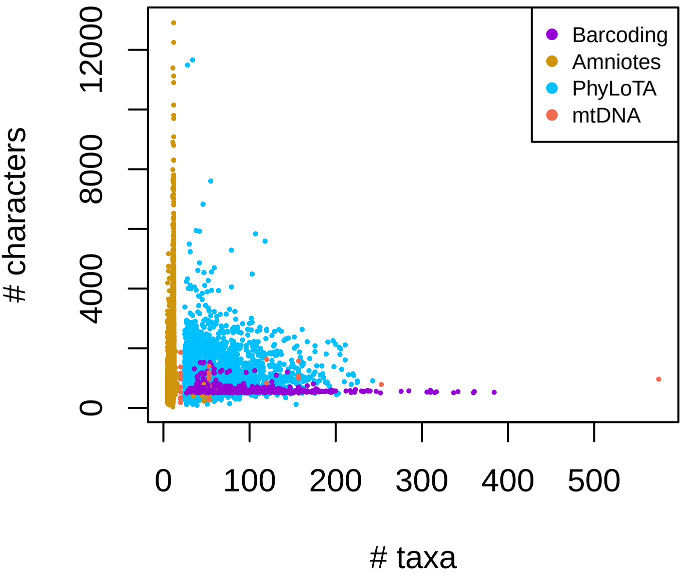

Barcoding
Amniotes
PhyLoTA
mtDNA
characters
0
4000
8000
12000
0
100
200
300
400
500
taxa

Table S1. MCMC performance diagnostics and dataset features. All diagnostics and acceptance rates are calculated after removing 25% burn-in from chains.

Feature
Parameter autocorrelation times
Parameter effective sample sizes (ESS)
Tree length
Number of generations to ESS of 200 for each parameter (multiples of 10% of chain length)
Topological approximate ESS
Topological autocorrelation time
Number of generations to topological ESS of 200 (multiples of 10% of chain length)
Average standard deviation of split frequencies (ASDSF) – no discard threshold
Correlation in split frequencies
Potential scale reduction factor (PSRF) for non-topology parameters
Correlations among parameters
Number of taxa
Number of characters
Standardized branch length in the tree
MCMC move acceptance rates
Acceptance rates of proposed swaps between cold and first heated chain

Table S2. Ratio of parameters that fail ESS per analysis in original analyses and after applying strategies to improve convergence following manual inspection of chains.

Analysis ID	original	run longer	HKY	Emp BF	Nst=Mixed
0000219ti=50622cl=2db=184	0.40	NA	0.00	0.00	0.37
0000241ti=4797cl=2db=184	0.72	NA	0.00	0.00	0.04
0000032ti=162262cl=7db=184	0.65	NA	0.00	0.00	0.00
0000035ti=92581cl=1db=184	0.78	NA	0.00	0.00	0.29
0000042ti=70899cl=7db=184	0.72	NA	0.00	0.00	0.76
0000049ti=186774cl=4db=184	0.52	NA	0.00	0.00	0.35
0000027ti=6964cl=7db=184	0.48	NA	0.00	NA	0.50
0000066ti=92558cl=12db=184	0.42	0.13	0.00	0.00	0.50
0000068ti=239225cl=3db=184	0.45	0.00	NA	NA	0.01
0000080ti=7402cl=0db=184	0.55	NA	0.40	0.00	0.10
0000085ti=64388cl=1db=184	0.52	NA	0.15	0.00	0.04
0000090ti=351514cl=4db=184	0.78	NA	0.00	0.00	0.00
0000105ti=70899cl=10db=184	0.68	NA	0.00	0.45	0.09
0000110ti=287187cl=35db=184	0.48	NA	0.00	0.00	0.22
0000112ti=50630cl=2db=184	0.62	0.00	0.18	0.00	0.71
0000141ti=360072cl=0db=184	0.87	NA	0.45	0.00	0.35
0000162ti=71526cl=1db=184	0.75	NA	0.00	0.00	0.18
0000174ti=243881cl=2db=184	0.43	NA	0.28	0.07	0.19
0000197ti=9526cl=47db=184	0.40	NA	0.30	0.18	0.19
0000209ti=27532cl=9db=184	0.47	0.07	NA	NA	0.04

Table S3. Number of diagnostic thresholds failed per analysis in original analyses and after applying strategies to improve convergence following manual inspection of chains.

Analysis ID	original	run longer	HKY	Emp BF	Nst=Mixed
0000219ti=50622cl=2db=184	16	NA	11	7	16
0000241ti=4797cl=2db=184	8	NA	4	4	5
0000032ti=162262cl=7db=184	11	NA	4	0	4
0000035ti=92581cl=1db=184	12	NA	8	4	12
0000042ti=70899cl=7db=184	12	NA	0	4	8
0000049ti=186774cl=4db=184	12	NA	4	4	12
0000027ti=6964cl=7db=184	12	NA	4	NA	12
0000066ti=92558cl=12db=184	12	9	8	4	11
0000068ti=239225cl=3db=184	8	0	NA	NA	5
0000080ti=7402cl=0db=184	8	NA	8	4	10
0000085ti=64388cl=1db=184	12	NA	6	4	5
0000090ti=351514cl=4db=184	12	NA	4	4	4
0000105ti=70899cl=10db=184	15	NA	7	10	12
0000110ti=287187cl=35db=184	11	NA	4	4	10
0000112ti=50630cl=2db=184	10	0	8	4	9
0000141ti=360072cl=0db=184	12	NA	10	4	11
0000162ti=71526cl=1db=184	16	NA	4	4	15
0000174ti=243881cl=2db=184	16	NA	16	14	15
0000197ti=9526cl=47db=184	20	NA	20	19	19
0000209ti=27532cl=9db=184	8	1	NA	NA	5

Table S4. Whether analyses pass or fail ASDSF in original analyses and after applying strategies to improve convergence following manual inspection of chains.

Analysis ID	original	run longer	HKY	Emp BF	Nst=Mixed
0000141ti=360072cl=0db=184	Failed	NA	Failed	Failed	Failed
0000035ti=92581cl=1db=184	Failed	NA	Failed	Failed	Failed
0000090ti=351514cl=4db=184	Failed	NA	Failed	Failed	Failed
0000162ti=71526cl=1db=184	Failed	NA	Failed	Failed	Failed
0000241ti=4797cl=2db=184	Failed	NA	Failed	Failed	Failed
0000042ti=70899cl=7db=184	Failed	NA	Pass	Failed	Failed
0000105ti=70899cl=10db=184	Failed	NA	Failed	Failed	Failed
0000032ti=162262cl=7db=184	Failed	NA	Failed	Pass	Failed
0000112ti=50630cl=2db=184	Failed	Pass	Failed	Failed	Failed
0000080ti=7402cl=0db=184	Failed	NA	Failed	Failed	Failed
0000049ti=186774cl=4db=184	Failed	NA	Failed	Failed	Failed
0000085ti=64388cl=1db=184	Failed	NA	Failed	Failed	Failed
0000027ti=6964cl=7db=184	Failed	NA	Failed	NA	Failed
0000110ti=287187cl=35db=184	Failed	NA	Failed	Failed	Failed
0000209ti=27532cl=9db=184	Failed	Pass	NA	NA	Failed
0000068ti=239225cl=3db=184	Failed	Pass	NA	NA	Failed
0000174ti=243881cl=2db=184	Failed	NA	Failed	Failed	Failed
0000066ti=92558cl=12db=184	Failed	Failed	Failed	Failed	Failed
0000219ti=50622cl=2db=184	Failed	NA	Failed	Failed	Failed
0000197ti=9526cl=47db=184	Failed	NA	Failed	Failed	Failed
